# A New Species of the Genus *Tylototriton* (Amphibia, Salamandridae) from Southwestern Hunan Province, China †

**DOI:** 10.3390/ani16152340

**Published:** 2026-07-31

**Authors:** Yi-Han Ma, Wen-Jing Deng, Na Zhang, Sun-Jun Xiang, Ya-Lan Xu, Le-Qiang Zhu, Hui Li, Jie Huang, Bin Wang, Sheng-Chao Shi, Xiao-Yang Mo

**Affiliations:** 1 Key Laboratory of Biodiversity Research and Utilization, College of Life Science, Hunan Normal University, Changsha 410081, China; 2 College of Biological and Food Engineering, Huaihua University, Huaihua 418000, China; 3 School of Environmental Resources, Changsha Environmental Protection Vocational College, Changsha 410004, China; 4 Wuhan Galsang Environmental Technology Co., Ltd., Wuhan 430080, China; 5 Hunan Engineering Laboratory for Giant Salamander Resource Conservation and Comprehensive Utilization, School of Life Sciences, Jishou University, Zhangjiajie 427000, China; 6 Hubei Engineering Research Center for Protection and Utilization of Special Biological Resources in the Hanjiang River Basin, School of Life Science, Jianghan University, Wuhan 430056, China

**Keywords:** *Tylototriton sheni*, molecular phylogeny, taxonomy, Salamandridae, Xuefeng Mountains

## Abstract

This study describes a new species of the newt genus (*Tylototriton sheni* sp. nov.) from the Huangyan area in Zhongfang County of Hunan Province, China, based on morphological and molecular genetic evidence. Phylogenetic analyses based on mitochondrial 16S rRNA and *ND2* gene sequences revealed that the new species forms a monophyletic clade with *T. maolanensis* and *T. broadoridgus*, all belonging to the *T. asperrimus* species group. The new species diagnosed by a combination of following characters: (1) Medium-sized body (SVL 60.9–70.1 mm in males and 74.6–75.5 mm in females); (2) Vomerine teeth in a wide inverted V shape, tips separated; (3) Tubercles on dorsolateral body disconnected, total munbers 16–18; (4) Head length greater than head width; (5) The posterolateral part of the squamosal bears a distinct process extending beyond the posterior margin of the exoccipital; (6) Tips of forelimbs and hindlimbs reaching and slightly overlapping when folded towards the body; (7) Tail shorter than body (TAL/SVL = 0.79–0.91 in males, 0.72–0.75 in females); (8) Fingertips extending beyond the tip of the snout when the forelimbs are stretched forward. This paper summarizes the developmental data of embryos of the new taxon. We propose that the conservation status of the new species should be Critically Endangered (CR) following the IUCN criteria (B1ab iii), and the new species should be included in the List of State Key Protected Wild Animals of China. Immediate protection and supervision of its population are recommended.

## Simple Summary

The genus *Tylototriton* belongs to the family Salamandridae within the order Urodela and is widely distributed across temperate and subtropical zones of the Northern Hemisphere. In previous field investigations, *Tylototriton* specimens collected from the Huangyan area of the Xuefeng Mountains in Hunan Province were assigned to the *T. wenxianensis* population. Recently, our phylogenetic and morphological analyses confirmed that the population in the southwestern Xuefeng Mountains represents an undescribed species. Herein, we name it *Tylototriton sheni* sp. nov., and present its detailed morphological description and taxonomic diagnosis.

## 1. Introduction

In 1871, Anderson established a new genus and named it *Tylototriton* (family Salamandridae), based on a specimen of a new caudata amphibian collected in Husa of Longchuan County and Tengchong, western Yunnan, China, and described the new species *T. verrucosus* [1]. Years later, Unterstein described another species, *T. asperrimus*, which was discovered in the Dayao Mountains of Jinxiu County, China [2]. Three new species of the genus *Tylototriton* were described between 1932 and 1984 [3,4,5]. With in-depth field investigation and the development of molecular research, more new species of *Tylototriton* have been described over the 21st century.

Currently, there are 53 known species of *Tylototriton* worldwide [6], with 25 species recorded in China [7]. These species distributed in China, arranged chronologically from oldest to most recently described, include: *T. verrucosus* (Aaderson, 1871), *T. asperrimus* (Unterstein, 1930), *T. kweichowensis* (Fang, et al., 1932), *T. taliangensis* (Liu, 1950), *T. hainanensis* (Fei, et al., 1984), *T. wenxianensis* (Fei, et al., 1984), *T. shanjing* (Nussbaum, et al., 1995), *T. dabienicus* (Chen, et al., 2010), *T. broadoridgus* (Shen, et al., 2012), *T. lizhenchangi* (Hou, et al., 2012), *T. pseudoverrucosus* (Hou, et al., 2012), *T. pulcherrimus* (Hou, et al., 2012), *T. yangi* (Hou, et al., 2012), *T. ziegleri* (Nishikawa, et al., 2013), *T. liuyangensis* (Yang, et al., 2014), *T. anhuiensis* (Qian, et al., 2017), *T. panwaensis* (Grismer, et al., 2019), *T. maolanensis* (Li, et al., 2020), *T. sini* (Lyu, et al., 2021), *T. daloushanensis* (Zhou, et al., 2022), *T. joe* (Rao, et al., 2022), *T. tongziensis* (Li, et al., 2022), *T. gaowangjienensis* (Huang, et al., 2024), *T. wufengensis* (Li, et al., 2026), and *T. guilinensis* (Xiao, et al., 2026) [1,2,3,4,5,8,9,10,11,12,13,14,15,16,17,18,19,20,21,22,23].

The origin and diversification of amphibians are thought to have arisen from a combination of geographic patterns and environmental changes. Salamanders of the genus *Tylototriton* are distributed over southern, eastern, and southeastern Asia, mainly throughout the eastern Himalayas and its extensions, including China, Vietnam, Laos, Nepal, Bhutan, Myanmar, Thailand, and India [9,10,11,14,15,24,25,26]. Moreover, this species is widely distributed throughout China, starting from Wenxian County of Gansu in the north, to Hainan in the south, and starting from the eastern side of the Hengduan Mountain in the west, to the Dabie Mountain in the east, including Gansu, Sichuan, Yunnan, Hunan, Anhui, Guizhou, Guangxi, and Hainan. Previous studies have suggested that the Hengduan Mountain region and the upper reaches of the Yangtze River represent the likely origin and main diversification center of *Tylototriton* in China [15,27,28]. This hypothesis was based on the genus’ distribution across inland China, which was interpreted as the result of northward and eastward population dispersal driven by river and mountain systems. Later, this suggestion was confirmed by Wang et al., who reported that the Northern Indochina Peninsula in southeastern Asia should be considered the origin and diffusion center of the genus *Tylototriton* [29].

Despite considerable progress in understanding the species diversity of *Tylototriton*, numerous cryptic species and species complexes within the genus remain to be clarified and formally described. Grismer et al. identified several distinct, undescribed lineages using molecular and morphological evidence, suggesting that these likely represent unnamed taxa [16]. Wang et al. further revealed that *T. wenxianensis* represents a species complex containing at least three cryptic lineages distributed in the Dabie Mountains of Anhui, Wufeng of Hubei, and Libo of Guizhou, respectively [29]. Based on integrated morphological and molecular phylogenetic analyses, the lineage from the Dabie Mountains was described as *T. anhuiensis* [15], and the lineage from Libo was named *T. maolanensis* [17]. The population from Wufeng was subsequently described as *T. wufengensis* [22]. Additional recently recognized species include *T. tongziensis* from the Dalou Mountains and *T. gaowangjienensis* from southwestern Hunan. Overall, the true species diversity in the complex mountainous regions of southern China remains severely underestimated [18,30,31].

During recent amphibian surveys in the Huangyan area (Zhongfang County, Huaihua City, Hunan Province), we encountered a *Tylototriton* population previously assigned to *T. wenxianensis* [32,33]. Integrating phylogenetic and morphological analyses with museum specimens of this population at Hunan Normal University, we confirm that the Huangyan population from the southwestern Xuefeng Mountains represents a candidate new species.

## 2. Materials and Methods

### 2.1. Specimens

**Figure 1:**
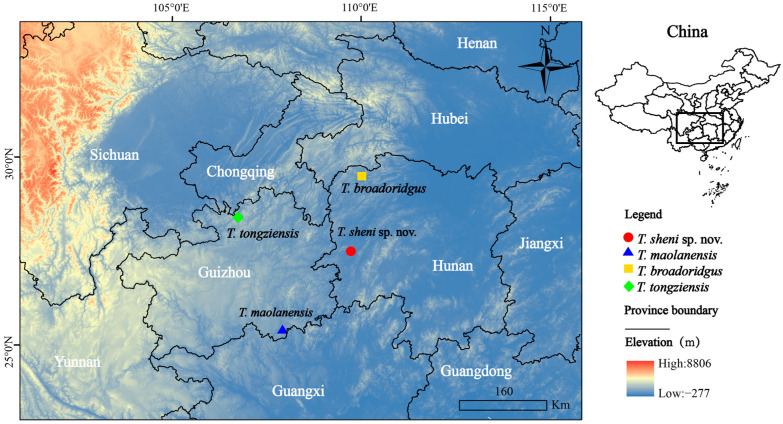
Collection localities of *Tylototriton sheni* sp. nov. from the Xuefeng Mountains, Hunan, China, and distribution of its three closely related species mentioned in this work.

Ten adult specimens (ten males) were collected in the Huangyan area of the southwestern Xuefeng Mountains, Hunan Province, China, in April 2026 (Figure 1). Specimens were fixed in 50% ethanol for 30 min and later preserved in 75% ethanol. All specimens were deposited in the College of Life Science, Hunan Normal University in Hunan, China.

After field collection, the specimens were euthanized under anesthesia. Muscle tissue samples were excised from each individual and preserved in 75% ethanol for molecular analyses. Whole specimens were first fixed in 50% ethanol for 30 min and then transferred to 75% ethanol for long-term preservation. All voucher specimens are deposited in the Animal Museum of the College of Life Sciences, Hunan Normal University (HUNL), Changsha, China. All procedures in this study were approved by the Animal Ethical and Welfare Committee of Hunan Normal University (Approval No. 2024829).

Furthermore, we examined specimens curated in the Animal Museum of Hunan Normal University. We examined seven specimens of the candidate new species (HUNL 2021042302, HUNL 201605007, 08, males; HUNL 201605005, 06, 35, HUNL 2021042301, females). For the congeneric species *T. maolanensis*, one specimens was examined (HUNL 2025061601, male), and, for *T. broadoridgus*, eight specimens were examined (HUNL 19840513513, 527, 529, 532, HUNL 1982-859, males; HUNL 840513606, HUNL 1988-V-2, V-3, females). All specimens were collected from their type locality (Appendix A).

### 2.2. Morphological Analysis and Skeleton Scanning

#### 2.2.1. Morphological Analyses

Morphological studies were conducted using a combination of descriptive and mensurative methods [20,22,24,34]. Morphological measurements were made with dial calipers to the nearest 0.01 mm. A total of 22 adult specimens were examined and measured for 20 morphometric characters, comprising 13 specimens of the candidate new species (10 males and 3 females), 1 specimen of *T. maolanensis* (male), and 8 specimens of *T. broadoridgus* from its type locality (5 males and 3 females). Additionally, morphological data for 8 *T. maolanensis* specimens (6 males and 2 females) were obtained from Li et al. [17], and 6 *T. tongziensis* specimens (4 males and 2 females) were obtained from Li et al. [20].

Abbreviated as follows: total length (TOL, distance from tip of snout to tip of tail); snout-vent length (SVL, distance from the tip of the snout to the posterior edge of the vent); head length (HDL, distance from the tip of the snout to the articulation of jaw); maximum head width (HDW, greatest width between the left and right articulations of the jaw); trunk length (TRL, from gular fold of throat to anterior tip of cloaca); snout length (SL, distance from the tip of the snout to the anterior corner of the eye); eye diameter (ED, distance from the anterior corner to the posterior corner of the eye); internasal distance (IND, minimum distance between the inner margins of the external nares); upper eyelid width (UEW, greatest width of the upper eyelid margins measured perpendicular to the anterior-posterior axis); interorbital distance (IOD, minimum distance between the inner edges of the upper eyelids); manus length (ML, distance from tip of third digit to proximal edge of inner palmar tubercle); length of lower arm (LLA, distance from elbow to tip of wrist); the third finger length (FIIIL, distance from base to tip of finger III); ibia length (TL, distance from knee to tarsi); the third toe length (TIIIL, distance from base to tip of toe III); tail length (TAL, from posterior margin of the anal opening to the tip of the tail); tail height (TH, maximum tail height); tail base width (TW, the maximum width between the lateral edges of the tail base at the level of the cloaca); width of dorsal ridge (DRW, the maximum width of the dorsal ridge); dorsal ridge tubercle number (DRN, number of tubercles along the dorsal ridge). All data are provided in Supplementary Material Table S1.

PCA was conducted to highlight species separation in morphometric space. The raw morphometric data were processed following the analytical protocol described by Huang et al.: all raw linear measurements were first natural log-transformed to normalise distributions and reduce heteroscedasticity, then adjusted to remove interspecific allometric body-size effects via the formula: X_a_ = X_ln_ − β⋅(SVL_ln_ − SVL_m_), where X_a_ = adjusted value; X_ln_ = ln-transformed measurements; β = unstandardized regression coefficient for each species; SVL_ln_ = ln-transformed SVL; and SVL_m_ = overall average SVL_ln_ of each species. We performed one-way ANOVA on the fully adjusted morphometric dataset to detect interspecific mean differences for each character (α = 0.05), with Levene’s test conducted prior to all ANOVA runs to verify homogeneity of variances (homoscedasticity accepted at *p* > 0.05). For traits yielding significant interspecific differences, post-hoc Tukey’s HSD tests were implemented to identify pairwise species divergence [35]. Statistical analyses were performed in R 4.5.1.

#### 2.2.2. Skeleton Scanning

Micro-CT scanning was performed on skeletal preparations of adult specimens, including six candidate new species (HUNL 201605005–08, HUNL 2021042301–02), 1 *T. maolanensis* specimen (HUNL 2025061601), and four *T. broadoridgus* specimens (HUNL 201605001–04). All specimens were collected from their respective type localities (Appendix A).

The skeletal scanning of candidate new species specimens HUNL 201605005, 06 (females), 07, 08 (males), and *T. broadoridgus* specimens HUNL 201605001, 02, 03 (males), and 04 (female) was conducted at the Chengdu Institute of Biology, Chinese Academy of Sciences, using a Quantum GX in vivo optical imaging system (PerkinElmer, Shelton, CT, USA). The scanning parameters were set to 70 kV (voltage), 80 μA (current), a view of 36 × 25, and high-resolution scan mode. The remaining three specimens were scanned at Central South University using a U-OIBLT/FLT/CTXUHR system (MILabs, Houten, The Netherlands), with parameters set to 55 kV (voltage), 0.17 mA (current), 1024 × 1024 pixels resolution, and high-resolution scan mode. Skeletal terminology follows the standards of Fei and Shen [34,36].

### 2.3. Molecular and Phylogenetic Analyses

Genomic DNA was extracted from the muscle tissue of seven field-collected specimens using a DNA extraction kit (Tiangen Biotech Co., Ltd., Beijing, China). Fragments of the mitochondrial 16S ribosomal RNA (16S rRNA) gene and NADH dehydrogenase subunit 2 (*ND2*) gene were amplified using the primer pairs P7 (5′-CGCCTGTTTACCAAAAACAT-3′)/P8 (5′-CCGGTCTGAACTCAGATCACGT-3′) and SL-1 (5′-ATAGAGGTTCAAACCCTCTC-3′)/SL-2 (5′-TTAAAGTGTCTGGGTTGCATTCAG-3′), respectively, following the protocols described in Simon et al. and Wang et al. [29,37]. The PCR conditions for the 16S rRNA gene were as follows: an initial denaturation at 95 °C for 4 min; 35 cycles of denaturation at 95 °C for 40 s, annealing at 52 °C for 40 s, and extension at 72 °C for 70 s; followed by a final extension at 72 °C for 10 min. For the *ND2* gene, the amplification profile consisted of an initial denaturation at 95 °C for 4 min; 35 cycles of denaturation at 95 °C for 40 s, annealing at 53 °C for 34 s, and extension at 72 °C for 60 s; followed by a final extension at 72 °C for 10 min. All PCR products were detected by 1% agarose gel electrophoresis and then sent to Shanghai DNA BioTechnologies Co., Ltd. (Shanghai, China) for sequencing. All newly obtained sequences were deposited in GenBank (Supplementary Material Table S2).

For phylogenetic analysis, 118 sequences from 38 recognized *Tylototriton* species (including the candidate new species) were downloaded and incorporated as the ingroup dataset [38,39,40,41,42,43,44,45,46]. Meanwhile, six sequences from three species in the Salamandridae family (*Echinotriton chinhaiensis* Chang 1932, *Pachytriton granulosus* Chang 1933, and *Pleurodeles waltl* Michahelles 1830) were selected and downloaded as outgroups (Supplementary Material Table S2). Sequences of the 16S rRNA and *ND2* genes were aligned using MEGA 12, followed by manual verification for accuracy [47]. Phylogenetic relationships were reconstructed based on the concatenated mitochondrial gene dataset using PhyloSuite v1.2.3. The best-fit evolutionary model for phylogenetic analysis was determined using ModelFinder. Maximum likelihood (ML) analysis was performed in IQ-TREE v2.2.0 [48]. The best-fit evolutionary model, TIM+F+I+G4, was selected by ModelFinder v2.2.0 under the Bayesian Information Criterion (BIC) [49]. Ultrafast bootstrap approximation (UFB) was assessed using 5000 ultrafast bootstrap replicates [50]. Bayesian inference (BI) analysis was implemented in MrBayes 3.6 [51], with the best-fit model GTR+F+I+G4. The Markov Chain Monte Carlo (MCMC) method was applied with four independent chains run for 5 million generations, sampling every 1000 generations. The first 25% of samples were discarded as burn-in. Nodes with Bayesian posterior probabilities (BPP) ≥ 0.95 were considered well-supported. Genetic distances among *Tylototriton* species were calculated using MEGA12 based on the uncorrected p-distance model for the 16S rRNA and *ND2* genes [47].

## 3. Results

### 3.1. Morphological and Skeleton Analyses

#### 3.1.1. Morphological Analyses

**Figure 2:**
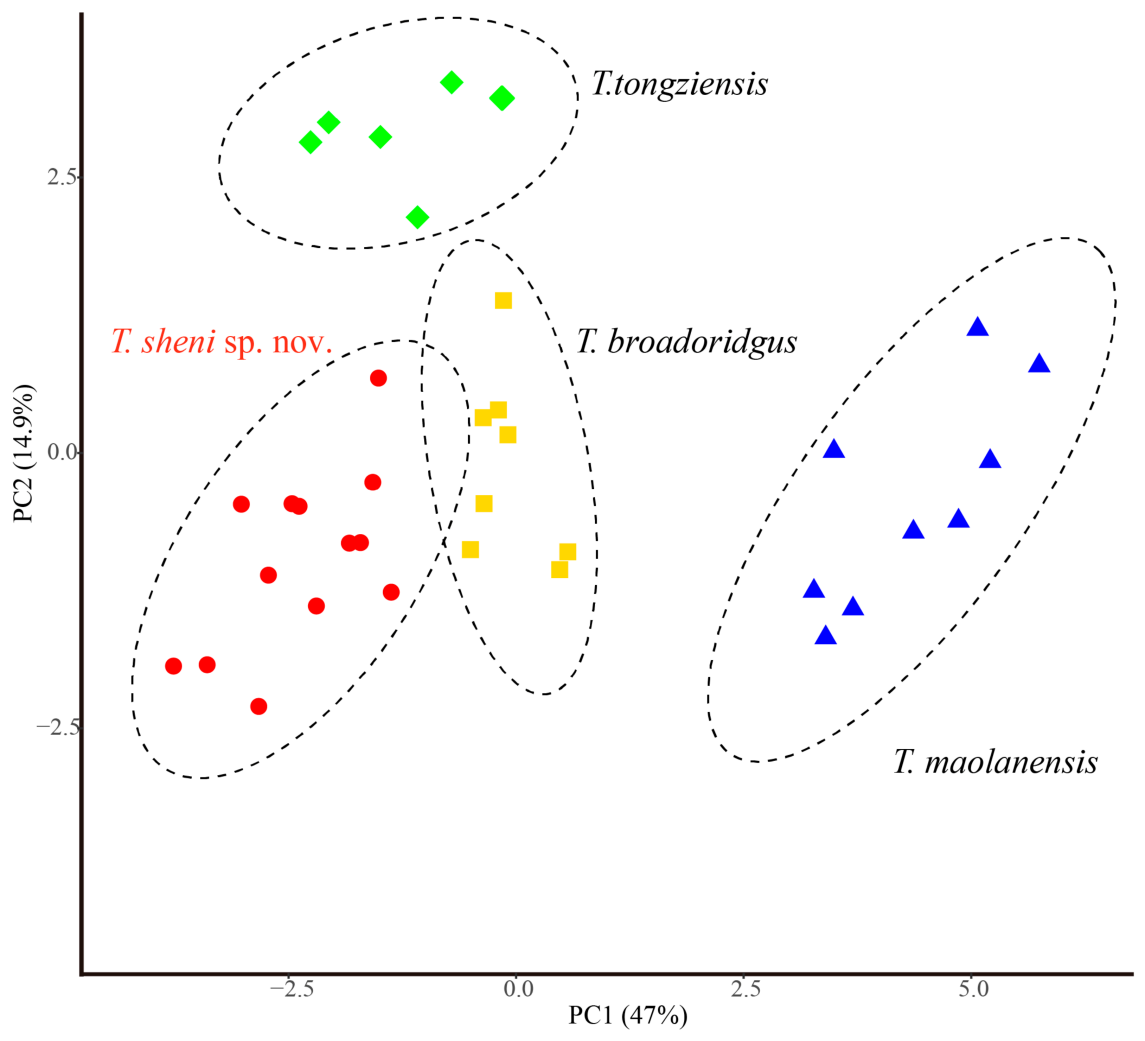
Plots of principal component analyses for *Tylototriton sheni* sp. nov. and its related species. PC1: the first principal component; PC2: the second principal component.

In PCA, the total variation of the first principal component was 47% and the second was 14.9% (Figure 2). One-way analysis of variance (ANOVA) further confirmed that adult males of the candidate new species differed significantly from the closely related species *T. maolanensis*, *T. broadoridgus*, and *T. tongziensis* in multiple morphometric characters, including SVL, HDL, HDW, TRL, ED, and IOD, et al. (all *p* < 0.05; see Supplementary Material Table S3 for details). Tukey’s HSD post hoc multiple comparisons revealed highly significant differences between the undescribed species and *T. maolanensis* in HDW, HDL, SL, and SVL, et al. (*p* < 0.05), as well as between the candidate new species and *T. broadoridgus* in HDW and TH (*p* < 0.05). Additionally, highly significant differences were detected between the candidate new species and *T. tongziensis* in IOD and TL (*p* < 0.05).

For females, one-way ANOVA results showed significant interspecific differences in characters including HDL, HDW, TRL, ED, and IOD, et al. (*p* < 0.05). Tukey’s HSD post hoc tests indicated significant differences between the candidate new species and *T. maolanensis* in HDL, HDW, and TRL (*p* < 0.05), between the candidate new species and *T. broadoridgus* in HDL and HDW (*p* < 0.05), and between the candidate new species and *T. tongziensis* in IOD and TL (*p* < 0.05). Notably, the level of significance for differences in most morphometric ratio characters was weaker in females than in males, suggesting sexual dimorphism in morphological traits (see Supplementary Material Table S3 for details).

#### 3.1.2. Skeleton Characteristics and Comparisons

**Figure 3:**
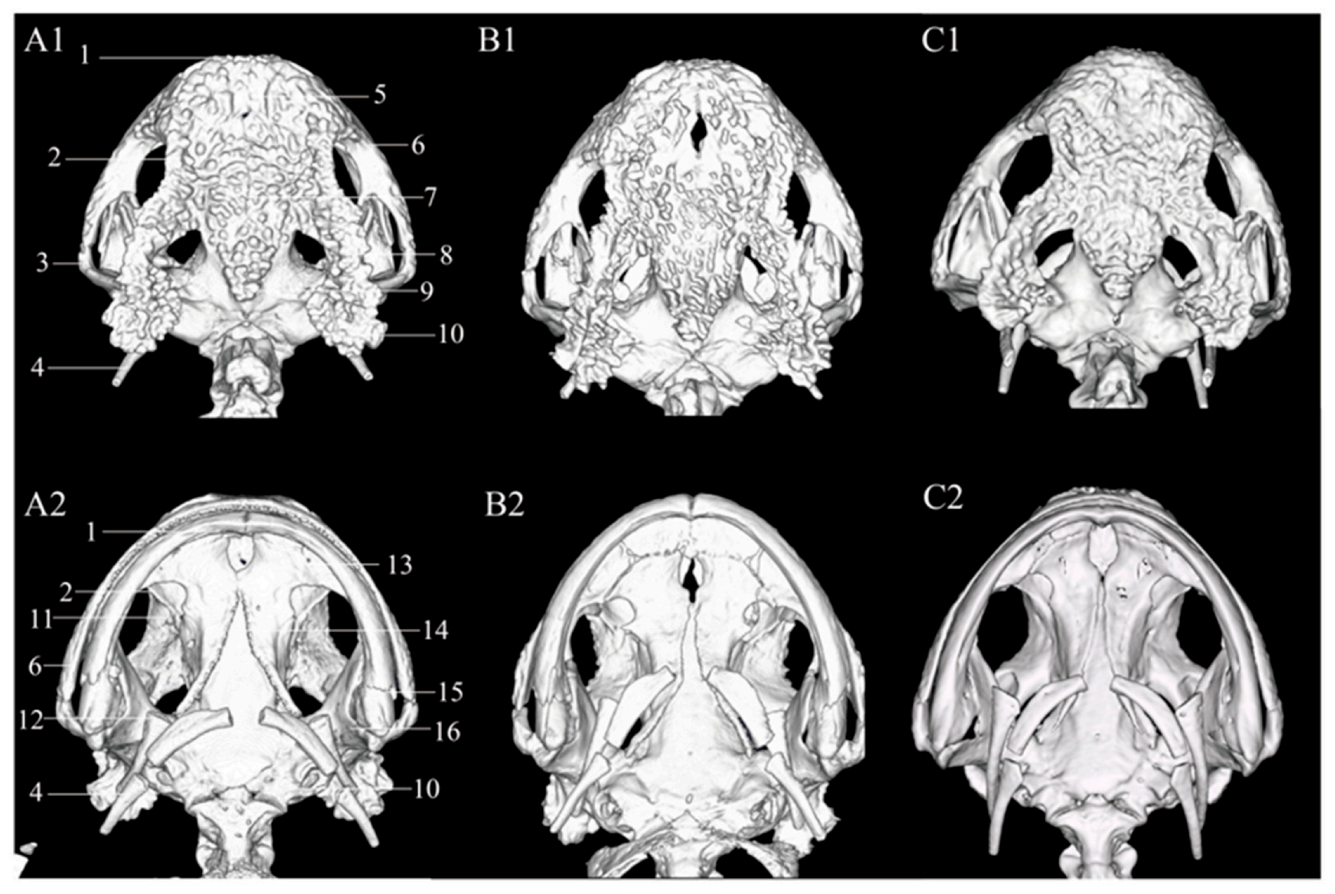
Skull morphological comparison of *Tylototriton sheni* sp. nov. and its two closely related species. (**A**) Skull (male paratype, HUNL 201605008) of *T. sheni* sp. nov.; (**B**) Skull (male topotype, HUNL 201605001) of *T. broadoridgus*; (**C**) Skull (male topotype, HUNL2025061601) of *T. maolanensis*; (**1**) dorsal view; (**2**) ventral view. (1) premaxilla; (2) prefrontal; (3) quadrate; (4) ceratobranchial 1; (5) nasal; (6) maxilla; (7) frontal; (8) squamosal; (9) parietal; (10) prootic-exoccipital complex; (11) orbitosphenoid; (12) ceratohyal; (13) vomer; (14) parasphenoid; (15) dentary; (16) pterygoid.

The skull morphology of different specimens of the candidate new species was almost identical, so one representative specimen (voucher number: HUNL 201605008) was selected for description (Figure 3(A1,A2)). The skull is longer than it is wide, with relatively large orbits and a broad, flat frontosquamosal arch. The premaxillae are paired, square-shaped, and located at the snout tip. The maxillae are paired, curved bones positioned laterally in the head; together with the premaxillae, they form the upper jaw. The anterior half of the maxilla articulates with the vomer to form the lateral wall of the nasal cavity, while its posterior end connects to the pterygoid.

The vomers are paired and fuse with the premaxillae and maxilla into a single bony unit. The two vomers form a wide angle, and their tooth rows do not meet anteriorly. They lie dorsal to the parasphenoid and extend posteriorly along the orbit to its posterior margin. The pterygoid is relatively flat and articulates with the maxilla. The parietal is small, raised into a roof-like shape, and tapers posteriorly to fit into the inner margin of the exoccipital. The squamosal bears a distinct spherical process posteriorly, extending beyond the posterior margin of the exoccipital. The fontanelle is small or absent.

The candidate new species exhibits consistent skull morphological differences from its closely related species *T. maolanensis* and *T. broadoridgus* (Figure 3(B1,B2,C1,C2)). In the candidate species, the cranial sutures are mostly indistinct, whereas they are clearly visible in both *T. maolanensis* and *T. broadoridgus*. The vomerine processes form a relatively wide anterior angle in the candidate new species, while this angle is smaller in *T. maolanensis*. The posterior end of the squamosal bears a distinct spherical process that extends beyond the posterior margin of the exoccipital in most specimens of the candidate new species; in contrast, the squamosal does not extend beyond the exoccipital in *T. maolanensis*, and although the squamosal projects posteriorly in *T. broadoridgus*, it does not form a distinct spherical process. The frontosquamosal arch is broad and flat in both candidate new species and *T. maolanensis* but narrow in *T. broadoridgus*. These characters allow for reliable differentiation among the three species.

### 3.2. Phylogenetic Analyses

**Figure 4:**
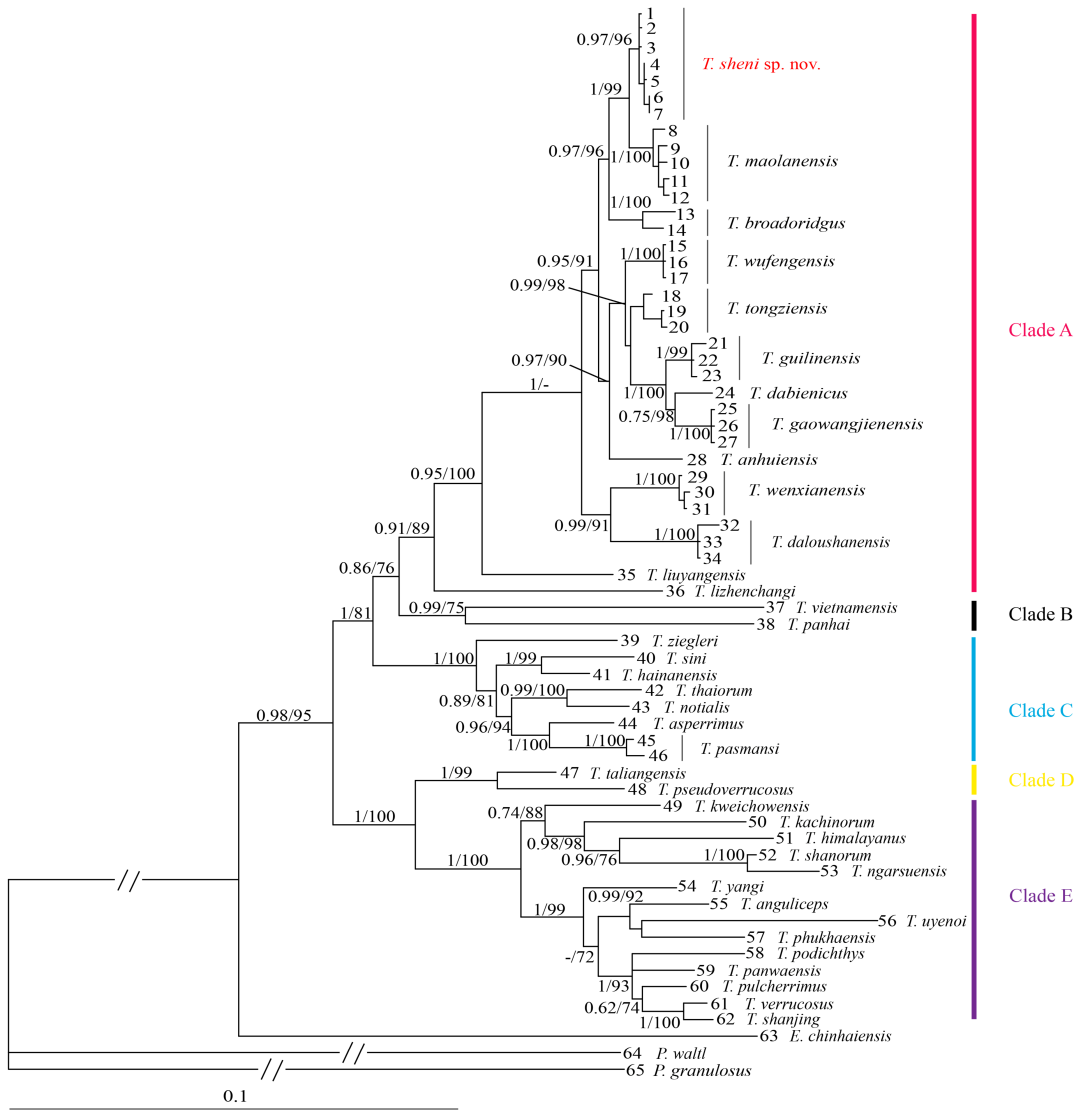
BI tree of the genus *Tylototriton* reconstructed based on the 16S and *ND2* gene sequences. Bayesian posterior probabilities/ML bootstrap supports are shown beside each node, and “-” denotes BS < 50% or BPP < 0.60. Samples 1–65 refer to Supplementary Material Table S2.

The aligned sequence lengths of the 16S rRNA and *ND2* genes were 509 bp and 1058 bp, respectively. Maximum likelihood (ML) and Bayesian inference (BI) analyses yielded congruent phylogenetic topologies, in which samples of the candidate new species formed a monophyletic clade with *T. maolanensis* and *T. broadoridgus* (Figure 4).

For the 16S rRNA gene, the uncorrected p-distances between the candidate new species and other congeners ranged from 0.60% to 6.86% (see Supplementary Material Table S4 for details). The genetic distances between the candidate new species and its closely related species, including *T. maolanensis*, *T. broadoridgus*, *T. tongziensis*, *T. wenxianensis*, *T. anhuiensis* and *T. liuyangensis*, were 0.60–0.80%, which were higher than or comparable to those observed between several known sister species pairs, such as *T. shanjing* and *T. verrucosus* (0.60%), *T. gaowangjienensis* and *T. tongziensis* (0.40%), and *T. wufengensis* and *T. tongziensis* (0.40%). For the *ND2* gene, the uncorrected p-distances between the candidate new species and other congeners ranged from 1.47% to 13.34% (see Supplementary Material Table S5 for details). The genetic distances between the candidate new species and its closely related species *T. maolanensis*, *T. broadoridgus* and *T. tongziensis* were 1.97% and 1.67%, respectively. These levels of differentiation were higher than or comparable to those observed between other known sister species pairs, including *T. sparreboomi* and *T. pasmansi* (0.49%), *T. verrucosus* and *T. shanjing* (1.18%), *T. gaowangjienensis* and *T. dabienicus* (1.67%), and *T. shanorum* and *T. ngarsuensis* (1.79%).

### 3.3. Morphological Comparison

**Figure 5:**
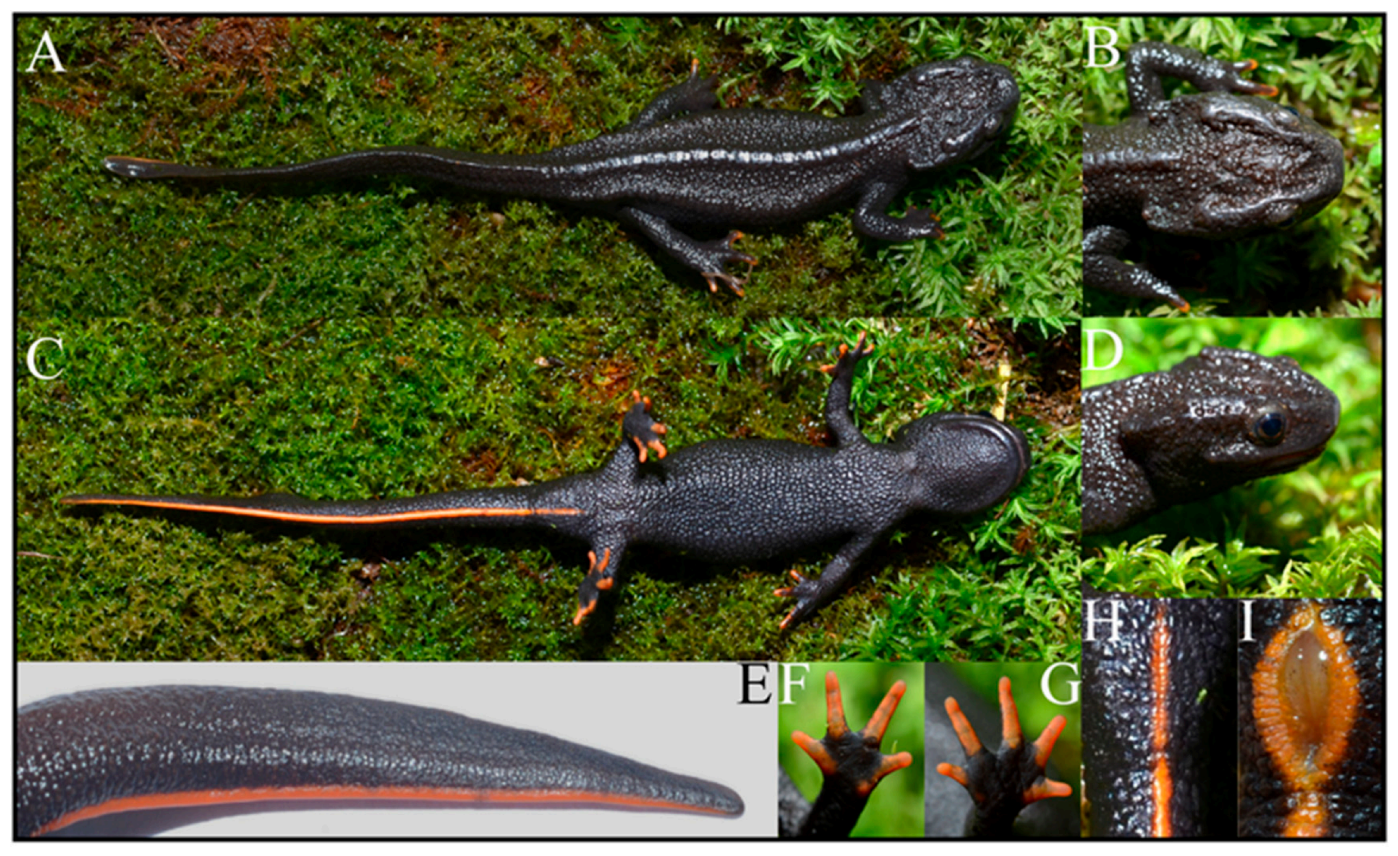
Holotype of *Tylototriton sheni* sp. nov. (male, HUNL 2026042402) in life. (**A**) Dorsal view; (**B**) Dorsal view of the head; (**C**) Ventral view; (**D**) Lateral view of the head; (**E**) Tail; (**F**) Ventral view of the hand; (**G**) Ventral view of the foot; (**H**) Closed cloaca; (**I**) Opened cloaca. (Photos by Yi-Han Ma).

**Figure 6:**
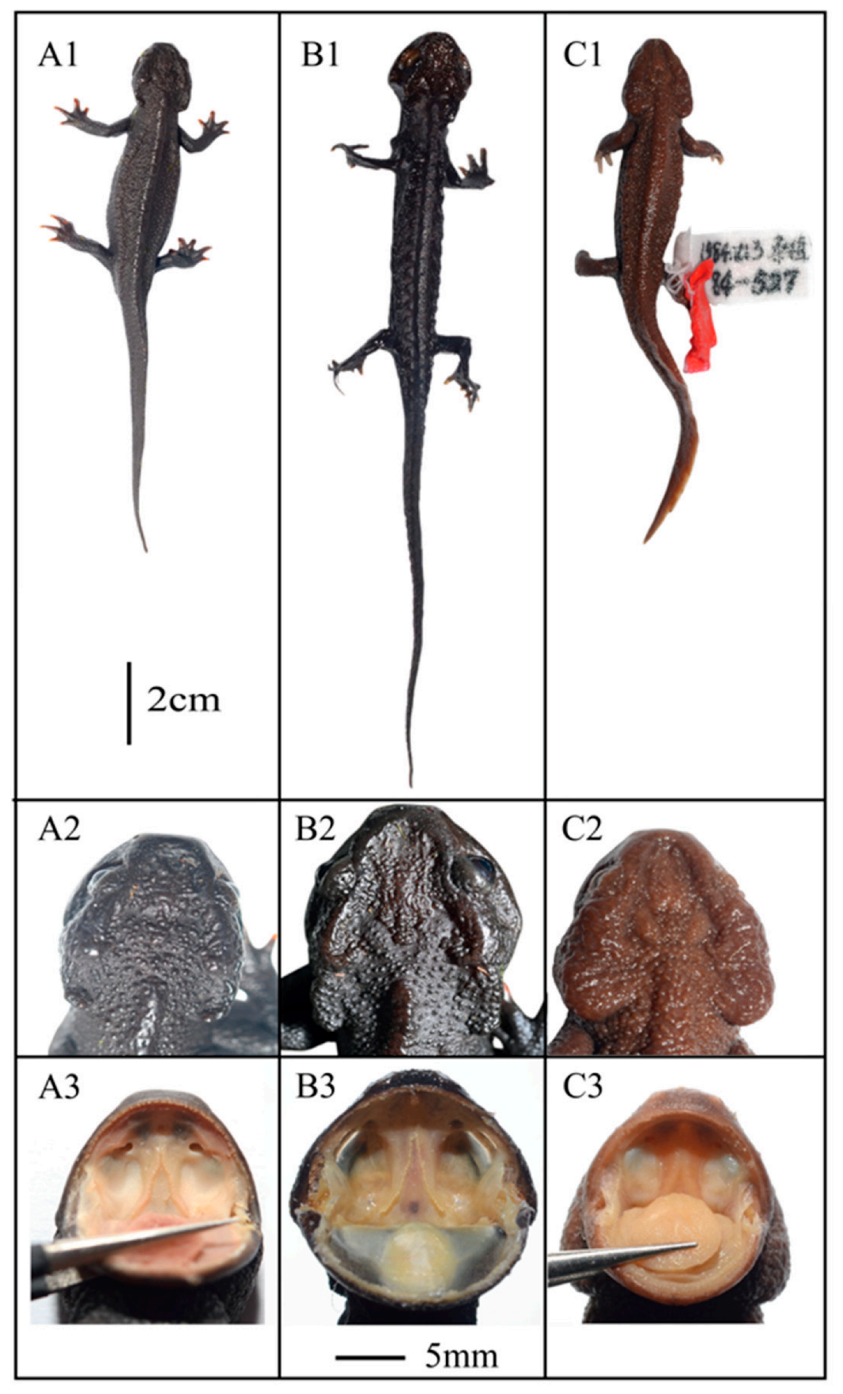
Holotype of *Tylototriton sheni* sp. nov. and specimens of its closely related species. (**A**) Holotype HUNL 2026042402; (**B**) Topotype HUNL 2025061601 of *T. maolanensis*; (**C**) Holotype HUNL 19840513527 of *T. broadoridgus*. (**A1**,**B1**,**C1**). Dorsal view of the whole body; (**A2**,**B2**,**C2**). Dorsal view of the head; (**A3**,**B3**,**C3**). View of the oral cavity. (Photos by Yi-Han Ma).

The Huangyan population has a series morphological characters matching of the genus *Tylototriton*, including rows of “˄” shaped vomerine teeth, a pair of prominent lateral bony ridges on the head, extremely rough dorsal and ventral skin covered with warts, a completely black body and tail, and a lack of an orange circular spot on the dorsal surface of the body (Figure 5 and Figure 6(A1–A3). Supplementary Material Table S6).

**Figure 7:**
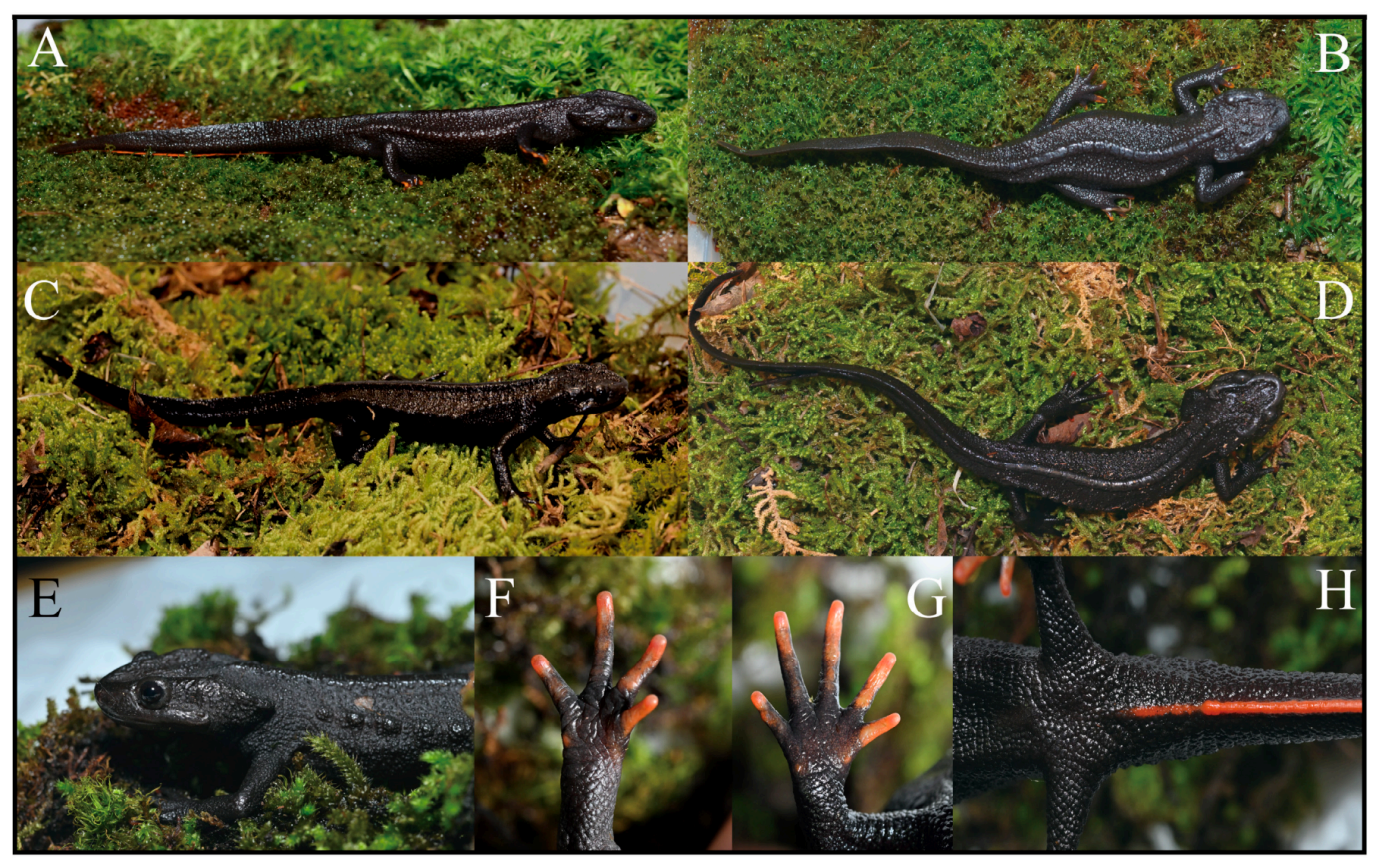
Holotype of the *Tylototriton sheni* sp. nov. and the topotype of *T. maolanensis.* (**A**,**B**) Holotype HUNL 2026042402, showing lateral view and dorsal view; (**C**–**H**) Topotype HUNL 2025061601. (**C**) Lateral view of the body; (**D**) Dorsal view; (**E**) Lateral view of the head; (**F**) Ventral view of the hand; (**G**) Ventral view of the foot; (**H**) Cloaca. (Photos **A**,**B** by Yi-Han Ma; Photos (**C**–**H**) by Hui Li).

The candidate new species can be distinguished from other congeners in the genus *Tylototriton* by the following combination of diagnostic morphological characters:

The candidate new species differs from *T. maolanensis* in: (1) medium-small body size, with SVL 60.9–70.1 mm in males (*n* = 6) and 74.6–75.5 mm in females (*n* = 7) VS. larger body size, SVL 76.8–91.8 mm in males (*n* = 7) and 76.3–87.4 mm in females (*n* = 2); (2) tips of digits slightly overlapping when limbs are adpressed to the trunk VS. strongly overlapping; (3) relatively shorter tail, (TAL/SVL = 0.79–0.91 in males, 0.72–0.75 in females; VS. relatively longer tail TAL/SVL = 1.00–1.14 in males, 0.88–0.96 in females); (4) no genital papillae in the male cloaca VS. present. One-way ANOVA revealed significant differences between the undescribed species and *T. maolanensis* in HDW, HDL, TRL, IOD, and TH (Figure 6(B1–B3) and Figure 7).

The candidate new species differs from *T. broadoridgus* in: (1) middorsal ridge narrow with distinct and countable nodules VS. ridge broad and thick with indistinct nodules; (2) tail height subequal to tail base width, TH/TW = 1.00 (*n* = 13) VS. tail height distinctly greater than tail base width, TH/TW = 1.25 (*n* = 8); (3) relative toe length III > IV > II > I > V VS. III > IV > II > V > I; (4) tail musculature weak with low dorsal fin fold VS. tail musculature robust with thin and high dorsal fin fold; (5) tail tip conical VS. rounded. One-way ANOVA revealed significant differences between the undescribed species and *T. broadoridgus* in HDL and HDW (Figure 6(C1–C3)).

The candidate new species differs from *T. tongziensis* in: (1) tips of digits slightly overlapping when limbs are adpressed to the trunk VS. strongly overlapping; (2) dorsal tail fin fold low VS. high; (3) tail height subequal to tail base width VS. greater than tail base width; (4) no genital papillae in the male cloaca VS. present. One-way ANOVA revealed significant differences between the undescribed species and *T. tongziensis* in IOD and TL.

The candidate new species differs from *T. liuyangensis* in: (1) medium-small body size, with SVL 60.9–70.1 mm in males and 74.6–75.5 mm in females VS. larger body size, SVL 64.2–82.0 mm in males and 80.8–88.0 mm in females; (2) middorsal ridge with distinct and countable nodules VS. indistinct nodules; (3) tips of digits slightly overlapping when limbs are adpressed to the trunk VS. not meeting; (4) fingertips extending distinctly beyond the snout when forelimbs are extended forward VS. reaching only to the eye level; (5) relative finger length III > II > IV > I VS. II > III > I > IV; (6) dorsal tail fin fold low VS. high.

The candidate new species differs from *T. wenxianensis* in: (1) no villiform genital papillae in the male cloaca VS. present; (2) middorsal ridge with distinct and countable nodules VS. indistinct nodules; (3) relative toe length III > IV > II > I > V VS. III > IV > II > V > I; (4) fingertips extending distinctly beyond the snout when forelimbs are extended forward VS. reaching the snout tip; (5) snout truncate in dorsal view VS. obtusely rounded; (6) vomerine teeth forming an inverted “∧” shape with separated tips VS. tips connected.

The candidate new species differs from *T. gaowangjienensis* in: (1) middorsal ridge with distinct and countable nodules VS. indistinct nodules; (2) snout nearly square and truncate in dorsal view VS. short and rounded; (3) tips of digits slightly overlapping when limbs are adpressed to the trunk VS. strongly overlapping.

The candidate new species differs from *T. wufengensis* in: (1) head length greater than head width VS. head width greater than head length; (2) middorsal ridge with distinct and countable nodules VS. indistinct nodules; (3) fingertips extending distinctly beyond the snout when forelimbs are extended forward VS. reaching the snout tip.

The candidate new species differs from *T. guilinensis* in: (1) head length greater than head width VS. head width greater than head length; (2) middorsal ridge with distinct and countable nodules VS. indistinct nodules; (3) tips of digits slightly overlapping when limbs are adpressed to the trunk VS. strongly overlapping; (4) no genital papillae in the male cloaca VS. present.

The candidate new species differs from *T. dabienicus* in: (1) gular fold present VS. absent; (2) tips of digits slightly overlapping when limbs are adpressed to the trunk VS. not meeting; (3) vomerine teeth forming an inverted “∧” shape with separated tips VS. tips connected; (4) fingertips meeting when limbs are adpressed to the trunk VS. not meeting.

The candidate new species differs from *T. daloushanensis* in: (1) fingertips extending distinctly beyond the snout when forelimbs are extended forward VS. reaching midway between eye and nostril; (2) no genital papillae in the male cloaca VS. present.

The candidate new species differs from *T. anhuiensis* in: (1) vomerine teeth forming an inverted “∧” shape with separated tips VS. tips connected; (2) no genital papillae in the male cloaca VS. present.

The undescribed species differs from *T. lizhenchangi* in: (1) head length greater than head width VS. head width greater than or subequal to head length; (2) relative toe length III > IV > II > I > V VS. III > IV > II > V > I; (3) nodule-like warts on the body sides that are indistinct VS. separated and distinct.

From species in Clades B and C, including *T. vietnamensis*, *T. panhai*, *T. ziegleri*, *T. sini*, *T. hainanensis*, *T. thaiorum*, *T. notialis*, *T. asperrimus*, *T. sparreboomi*, and *T. pasmansi*, the candidate new species differs in having continuous and indistinct nodular warts on the flanks VS. discrete and prominent nodules.

From species in Clade D, the candidate new species differs from *T. taliangensis* and *T. pseudoverrucosus* in: (1) smaller body size, with TOL 117.0–127.0 mm in males and 114.1–135.2 mm in females VS. much larger size in *T. taliangensis* (males 186.0–220.0 mm, females 194.0–230.0 mm) and *T. pseudoverrucosus* (males 156.2–173.0 mm, female 178.2 mm); (2) tail length shorter than snout–cloaca length VS. longer; (3) body uniformly black except for orange on digit tips, cloacal periphery, and ventral tail margin VS. bright orange parotoid patches in *T. taliangensis* and bright reddish-brown markings on the head, dorsum, and tail margins in *T. pseudoverrucosus*.

From species in Clade E, including *T. anguliceps*, *T. himalayanus*, *T. houi*, *T. kachinorum*, *T. kweichowensis*, *T. ngarsuensis*, *T. ngoclinhensis*, *T. panwaensis*, *T. phukhaensis*, *T. podichthys*, *T. pulcherrimus*, *T. shanjing*, *T. shanorum*, *T. soimalai*, *T. umphangensis*, *T. uyenoi*, *T. verrucosus*, *T. yangi*, and *T. zaimeng*, the candidate new species differs in body uniformly black except for orange on digit tips, cloacal periphery, and ventral tail margin VS. bright reddish-brown markings on the head, dorsum, and tail margins.

### 3.4. Taxonomic Accounts

The results from the morphological and molecular systematic comparisons support that the population from the Huangyan area of the Xuefeng Mountains should be recognized as an undescribed species.

*Tylototriton sheni* Ma, Shi & Mo, sp. nov.

**Figure 8:**
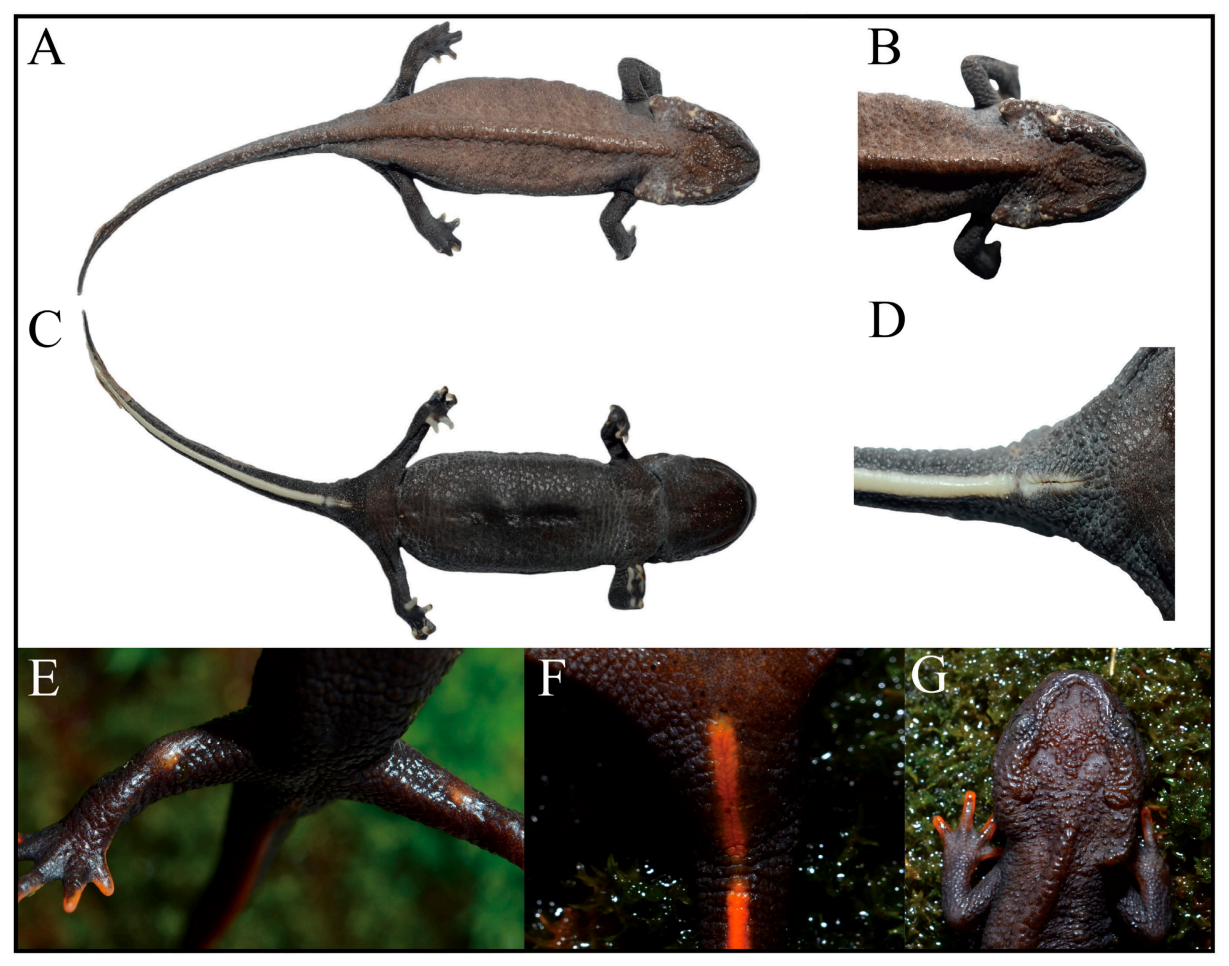
A female paratype of *Tylototriton sheni* sp. nov. (HUNL 2021042301) and Color variation in *T, sheni* sp. nov. (**A**) Lateral view; (**B**) Dorsal view of the head; (**C**) Ventral view; (**D**) closed cloaca. (**E**–**G**) Variation in *T. sheni* sp. nov. (**E**) Knee with orange blotch (HUNL2026042408); (**F**) Cloaca (HUNL2026042409); (**G**) Dorsal view of the head (HUNL2026042409) (Photos by Yi-Han Ma).

**Figure 9:**
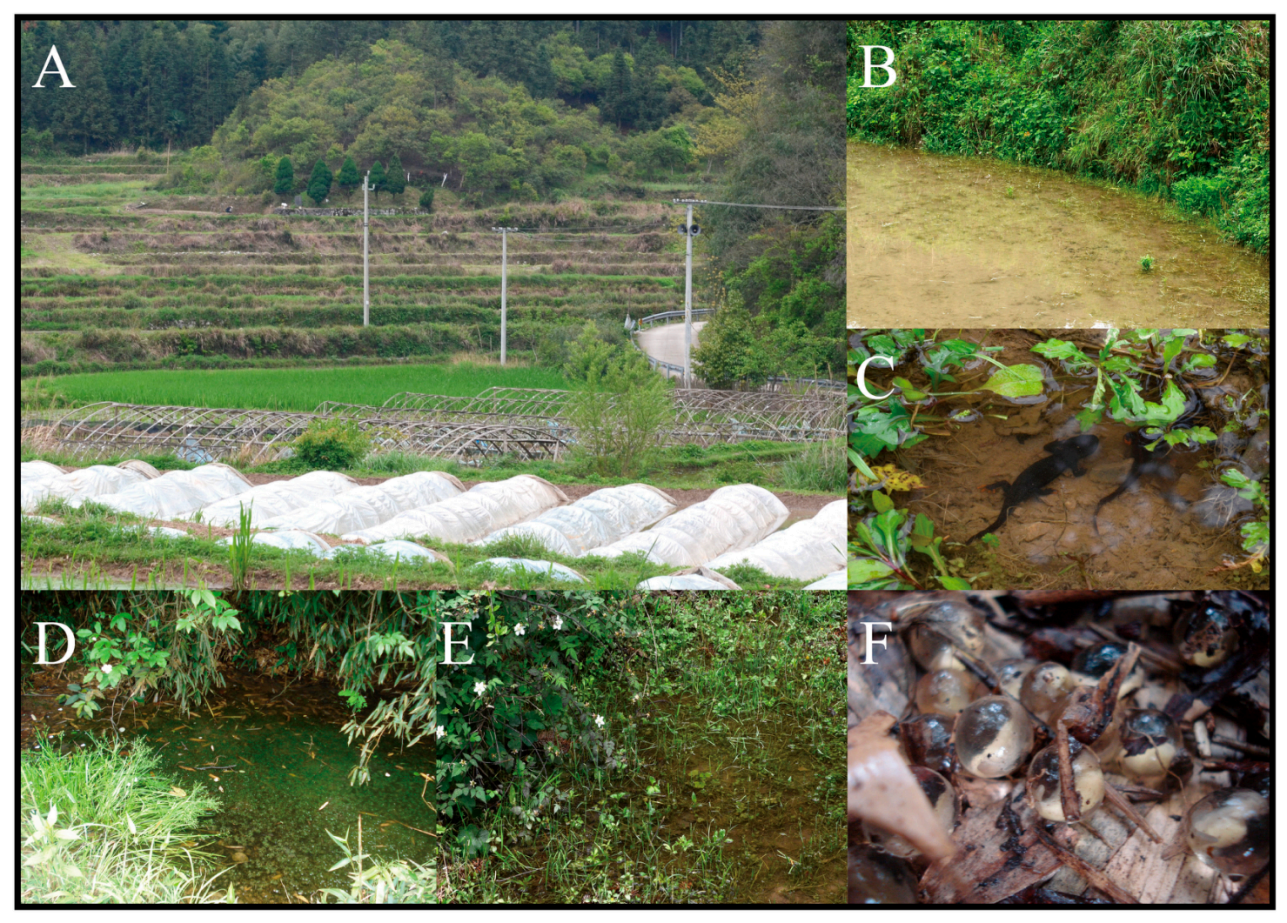
Habitats of *Tylototriton sheni* sp. nov. in the type locality, Zhongfang County, Huaihua City, Hunan Province, China. (**A**) Landscape; (**B**) Paddy field breeding puddles; (**C**) *T. sheni* sp. nov. in still-water pools; (**D**,**E**) Natural breeding puddles; (**F**) Egg mass. (Photos (**A**–**C**,**E**) by Yi-Han Ma in 2026; Photos **D**,**F** by Sheng-Chao Shi in 2012).

Figure 3, Figure 5, Figure 6, Figure 7, Figure 8 and Figure 9.

https://zoobank.org/FAC1D7EF-2833-43CC-80E6-98F2CD3E656C

https://zoobank.org/38FA756C-4C75-4DF5-9998-E8DC691C14A6

Chresonymy. *Tylototriton wenxianensis*-Xiang, et al., 2010a, 2010b [32,33].

**Holotype:** HUNL 2026042402, adult male, SVL 64.1 mm, was collected by Yi-Han Ma, Ya-Lan Xu, Le-Qiang Zhu, and Shen Jiang from the Xuefeng Mountains (27°28′ N, 110°04′ E, at an elevation of 852 m a. s. l.) in Zhongfang County, Hunan on 24 April 2026.

**Paratypes:** (*n* = 12) HUNL 2026042401, 03-10 were collected by the same people from the same place at the same time. HUNL 2021042301 was collected by You-Xiang Zhang and Tao Wu on 23 April 2021. HUNL 20160505, 35, were collected by Xiao-Yang Mo on 2 April 2016. All of the paratypes were collected from the same place as the holotype.

**Diagnosis:** *Tylototriton sheni* sp. nov. is assigned to the genus *Tylototriton* sensu lato based upon molecular phylogenetic analyses and the following morphological characters: presence of dorsal granules, dorsolateral bony ridges on the head, knob-like warts on the dorsolateral body, and absence of a quadrate spine. *T. sheni* sp. nov. can be distinguished from its congeners by the following combination of characters: (1) Medium-sized body (SVL 60.9–70.1 mm in males and 74.6–75.5 mm in females); (2) Vomerine teeth in a wide inverted V shape, tips separated; (3) Tubercles on dorsolateral body disconnected, total nunbers 16–18; (4) Head length greater than head width; (5) The posterolateral part of the squamosal bears a distinct process extending beyond the posterior margin of the exoccipital; (6) Tips of forelimbs and hindlimbs reaching and slightly overlapping when folded towards the body; (7) Tail shorter than body (TAL/SVL = 0.79–0.91 in males, 0.72–0.75 in females); (8) Fingertips extending beyond the tip of the snout when the forelimbs are stretched forward.

Description of the holotype (HUNL 2026042402): measurements in mm. Figure 5. Adult male, TOL 121.1 mm, SVL 64.1 mm (see Supplementary Material Table S1 for details). The head is comparatively high, with the top slightly concave; head length is longer than head width (HDL/HDW = 1.06). The snout is nearly square and extends beyond the lower lip, appearing truncated in dorsal view. The dorsolateral bony ridges on the head are notably prominent and protrude clearly backward, extending from above the snout through the upper eyelid to the occiput. The two triangular bony ridges on the lateral occiput form a “∨” shape and are indistinctly connected to the dorsal ridge of the body. The internasal space is smaller than the interorbital space; eyes protrude from the dorsolateral portion of the head and are surrounded by ring eyelids. The oral fissure is flat and straight, extending more than half the length of the head and posterior to the caudal margin of the eyes; the jaw articulation lies posterior to the eyes. Fine teeth are present on the edge of the jaw, and the vomerine teeth row forms a “∧” shape, with the tips separated from each other. The tongue is oval, slightly concave in the middle, nearly entirely fixed at the base but free at both lateral edges. The neck is rounded and thick, with a distinct gular fold, and the edges of the groove are curved and smooth.

The body is stout. The glandular vertebral ridge is distinct, extending from the neck to the base of the tail, with a rough and segmented surface; the knob-like dorsal warts on the ridge are countable, with 16 distinct warts. No obvious small glands are present on the lateral body skin, and the rib nodules are indistinct. The ventral area of the body is relatively flat and smooth, with fine transverse striae between the warts.

The four limbs are slender, with hind limbs slightly longer than forelimbs. When the fore and hindlimbs are adpressed along the trunk, the tips of the digits slightly touch each other. When the forelimbs are stretched forward, the fingertips extend beyond the snout. There are four fingers on the forelimbs, with the relative length: III > II > IV > I. There are five toes on the hindlimbs, with the relative length: III > IV > II > I > V. All digits are slightly flat with obtuse ends and are without webbing.

The tail length is distinctly shorter than the snout–cloaca length. The tail is notably compressed laterally, with relatively emaciated tail muscles; the width of the tail base is 7.78 mm. The dorsal fin fold of the tail starts from the tail base and is distinctly thin and high, with fine vertical striae. The distal tail tip is conoid. The cloaca is long and narrow, with a fissure shape, and the cloacal region is slightly bulbous.

The skin is rough, covered with small tubercles. The labial margin, distal limbs, ventral limbs, and ventral edge of the tail are smooth. The tubercles on the lateral dorsum of the body are small, arranged in distinct lines extending from the shoulder to the base of the tail, with the nodule-like tubercles forming continuous rows and no clear separation between neighboring tubercles. The pharyngeal tubercles are small, distinct, and sparse, while the ventral tubercles and warts are relatively flat and smaller.

**Color in life.** In life, the overall body color of the specimen is nearly black or blackish-brown. The ventral fin fold of the tail, the peripheral area of the cloaca, the distal digit ends, and the ventral digits are all orange. The orange regions between the ventral fin fold of the tail and the peripheral area of the cloaca are connected (Figure 5, Figure 7A,B and Figure 8A–G).

**Color in preservative.** The specimen in preservative is nearly black or blackish-brown. The orange coloration of the distal digit ends, ventral digits, the peripheral area of the cloaca, and the ventral edge of the tail fades to light orange or lacteal.

**Variation.** Most specimens are morphologically similar to the holotype, but some individuals differ: an orange-red protrusion is visible behind the parotoid gland in some individuals; the knee joint is orange in some individuals (Figure 8E); the orange regions between the ventral fin fold of the tail and the peripheral area of the cloaca are disconnected in some individuals (Figure 8F), the left and right parotoid gland are asymmetrical in some individuals (Figure 8G).

**Secondary sexual characteristics and reproduction.** The cloacal fissure of males is relatively long (Figure 5H,I). The cloacal fissure of females is short, and the grooves on the inner wall extend radially from the center to the surrounding areas (Figure 8D). No villous papillae were found within the cloacal fissures of both males and females during the breeding season.

**Ecology and natural history.** From 2007 to 2010, Xiang et al. conducted continuous field monitoring of *Tylototriton* species in the Huangyan region [32,33]. They found that the breeding season of the Huangyan population distributed in Huaihua lasts from April to June, with the reproductive peak occurring between late April and early May. The population ends hibernation in late March. In early April, males begin to migrate to the breeding grounds and enter the breeding season; females arrive about one week later than males. In late May, females leave the breeding grounds after oviposition, while males remain for a period of time. By early June, all adults leave the breeding grounds completely and return to terrestrial habitats, marking the end of the breeding season. Survey data collected in 2016, 2021, and 2026 all corroborate the above-mentioned reproductive habits (Figure 9).

**Larva.** Embryonic development observations and research on larvae were conducted by Xiang et al. [32,33]. During 2008–2009, researchers sequentially observed the embryonic development of 182 fertilized eggs from four batches and statistically analyzed the developmental duration of embryos in eight culture dishes. The results revealed that, under a room temperature of 19–21 °C, the average total developmental duration of early embryos was (513.30 ± 7.93) h (*n* = 8), equivalent to 21.4 days. The detailed developmental timeline is shown in Table 1.

Larvae that had just hatched are full of black spots on the head, back, and both sides of the forelimbs, while the thorax and abdomen are yellowish white. The larvae have three pairs of external gills, but no balancer. The dorsal fin fold is well developed, from the area between the posterior head and the anterior body, and the ventral fin fold starts from the base of the tail. Most forelimbs have developed three fingers, while a few have only two. The hindlimbs are short and weak with two-finger differentiation.

**Table 1:** The embryonic development process of *Tylototriton sheni* sp. nov. [33].

Number	Developmental Time	Stage Developmental Time (*n* = 8)	Total Developmental Time
1	Fertilized egg	8.06 ± 0.22	8.06
2	2-cell stage	2.11 ± 0.19	10.17
3	4-cell stage	2.07 ± 0.29	12.24
4	8-cell stage	1.58 ± 0.14	13.83
5	16-cell stage	1.18 ± 0.14	15.01
6	Multi-cell stage	3.80 ± 0.33	18.81
7	Early blastula stage	3.74 ± 0.37	22.55
8	Late blastula stage	10.07 ± 0.65	32.62
9	Early gastrula stage	3.55 ± 0.58	36.17
10	Middle gastrula stage	2.01 ± 0.06	38.18
11	Late gastrula stage	11.33 ± 0.70	49.50
12	Neural plate stage	2.33 ± 0.59	51.83
13	Neural fold stage	3.48 ± 0.32	55.31
14	Neural groove stage	3.79 ± 0.37	59.10
15	Neural tube stage	6.32 ± 0.93	65.42
16	Early tail bud stage	39.08 ± 2.44	104.50
17	Late tail bud stage	54.68 ± 1.78	159.18
18	Early external gill stage	38.82 ± 3.08	198.00
19	Late external gill stage	36.68 ± 2.07	234.68
20	Early fore-limb bud stage	148.56 ± 2.72	383.24
21	Incubation stage	130.06 ± 12.49	513.30 ± 7.93

**Ecology.** *Tylototriton sheni* sp. nov. lives in a mountainous area at an elevation from 800 m to 1200 m. They generally live on land, which is covered by thick, dry branches and fallen leaves. During the breeding season, individuals enter almost still pools with clear water that are rich in humus at the bottom (Figure 9).

**Conservation Status.** Prior to 2012, the population of this species remained relatively abundant, yet subsequent field surveys have documented continuous population declines. All known localities of the species are highly clustered. Based on repeated survey records, its area of occupancy (AOO) is inferred to be less than 10 km^2^, while its extent of occurrence (EOO), modelled from contour lines using ArcMap, is estimated at 118 km^2^. Ongoing declines in the overall population have been consistently detected across all field expeditions. Field surveys and analyses identify two key threatening factors: first, concrete canal construction fragments contiguous habitats; these hardened channels also act as artificial traps where numerous adult *Tylototriton* individuals fall in and perish. Second, widespread abandonment of paddy fields drastically reduces breeding habitats available to the species. Given its extremely restricted range, continuously declining population, and severely threatened habitats, this taxon satisfies the IUCN Red List criterion B1ab (iii) for Critically Endangered (CR) [52]. We herein propose its conservation status as Critically Endangered and recommend its inclusion in the List of State Key Protected Wild Animals of China.

**Etymology.** The specific epithet sheni is named in honour of Professor Youhui Shen (沈猷慧), a Chinese zoologist who has made significant contributions to the study of Chinese amphibians and reptiles. In 2016, at the age of 84, Professor Shen was the first to hypothesize that this population might represent an undescribed species during a field survey in the Huangyan area of Huaihua. For the common name, we suggest “Huang Yan Knobby Newt” (English) and Huang Yan You Yuan (黄岩疣螈, Chinese).

## 4. Discussion

Combined molecular and morphological evidence confirms that this species possesses a suite of diagnostic characters consistent with *Tylototriton*, including a chevron-shaped vomerine tooth row, prominent paired lateral bony ridges on the head, rough and warty skin on both the dorsum and ventrum, and entirely black coloration. Nevertheless, it exhibits clear morphological and genetic distinctness from all known species within the *Tylototriton*. To date, four species of *Tylototriton* have been documented in Hunan Province [10,11,14,21]. Molecular phylogenetic analyses reveal that the Huangyan population is most closely related to *T. maolanensis* and *T. broadoridgus*, and together they form a late-diverging subclade within the *Tylototriton verrucosus* lineage. This population also exhibits high genetic similarity to *T. tongziensis* and *T. liuyangensis*. These species display distinct distribution patterns: *T. maolanensis* mainly occurs in the northern Nanling Mountains; *T. broadoridgus* is primarily found in the northern Wuling Mountains; *T. tongziensis* is distributed across the central Dalou Mountains; and *T. liuyangensis* inhabits the upper reaches of the Liuyang River.

According to Yao et al., the region from the Dabie Mountains to the Huaihe River likely serves as a secondary center of distribution and specialization for most species in this clade [28]. A lineage of *Tylototriton* probably first colonized inland China, then dispersed eastward along the Hengduan Mountains and the Jialing River system (including the Bailong River, a tributary of the upper Yangtze River) toward the Qinling Mountains. While some species (e.g., *T. wenxianensis*) established breeding populations in Gansu Province, others continued eastward along the Yangtze River and the Qinling Mountains. However, the geographical barrier formed by the Qinling and Dabie Mountains [53] drove lineage divergence: one group continued eastward along the Bashui and Wan rivers toward the Dabie Mountains, while another dispersed to the Dongting Lake area southeast of the Qinling Mountains. The complex river systems in the lake basin further promoted isolation: some populations migrated along the Lishui and Yuan rivers toward the Xuefeng Mountains, while others spread along the Xiang and Liuyang rivers toward the Dawei Mountains.

From a biogeographic perspective, dispersal and geographic isolation operated concurrently throughout the evolutionary process. Long-standing mountain barriers such as the Xuefeng Mountains and Dalou Mountains impeded gene flow between isolated populations. The long-term geographic separation of the Huangyan population in Huaihua has resulted in stable differentiation from its closely related congeners and the previously misidentified *T. wenxianensis* in historical records, with conspicuous interspecific differences in morphometric traits, reproductive behaviours and embryonic development [32,33]. Such speciation is inferred to be driven by prolonged geographic isolation. Mountain fragmentation within this region has created disjunct distributions of closely related species, which corroborates the synchronous occurrence of vicariant differentiation and historical dispersal. Thus, the observed discordance between genetic and geographical distances is likely the result of repeated isolation by mountains and rivers.

Field observations conducted between 2012 and 2026 show that the confirmed distribution sites of *Tylototriton sheni* sp. nov. are located in close proximity to human settlements. One of its primary breeding habitats is rice paddies, and individuals have even been found in clear, slow-flowing roadside ditches within villages during the breeding season. These habitats are facing increasing threats from human activities. It remains unclear whether this habitat selection is an active adaptation or a passive response to rapid environmental changes [54]. The extent of occurrence is estimated to be less than 100 km^2^, it is known from only a single location, and there is a continuing decline in habitat quality. Given that this taxon meets IUCN Red List criterion B1ab(iii) for Critically Endangered (CR), we herein propose its conservation status as Critically Endangered [52]. Furthermore, we recommend that the new species be included in the List of State Key Protected Wild Animals of China. Therefore, against the backdrop of ongoing environmental change, urgent additional surveys and long-term monitoring are needed to better understand and protect this species.

The present study conducts comprehensive, detailed comparisons between *Tylototriton sheni* sp. nov. and its closely related congeners, integratively delimiting this new taxon based on external morphological traits, osteological characters, and mitochondrial phylogenetic evidence. We further evaluate the distribution range and conservation status of this novel species. Owing to the limited phylogenetic information captured by a small set of nuclear gene fragments [29], we reconstructed phylogenetic trees using only mitochondrial 16S rRNA and *ND2* genes in this work. For follow-up investigations, we plan to incorporate a broader suite of molecular markers into phylogenetic analyses to retrieve richer genetic divergence data for robust species delimitation. In addition, the currently documented geographic distribution of *T*. *sheni* sp. nov. is narrow. Extensive large-scale field surveys are urgently required to fully clarify its complete distribution range.

## 5. Conclusions

The *Tylototriton* species distributed in the Huangyan area of the Xuefeng Mountains, Hunan Province, was previously identified as *T. wenxianensis.* Morphological and phylogenetic analyses of specimens collected from this area between 2012 and 2026 revealed that this population represents a new species, *Tylototriton sheni* sp. nov. After comprehensive assessment, we propose the conservation status of *T. sheni* as Critically Endangered (CR). We also recommend its inclusion in the List of State Key Protected Wild Animals of China and urge immediate population surveys and targeted conservation actions for this species.

## Data Availability

The original contributions presented in this study are included in the article/Supplementary Materials; further inquiries can be directed to the corresponding authors.

## Supplementary Materials

The following supporting information can be downloaded at: https://www.mdpi.com/article/10.3390/ani16152340/s1, Table S1: Comparative morphological data of *Tylototriton sheni* sp. nov. and related species; Table S2: Information for samples used in molecular phylogenetic analyses in this study; Table S3: Measurements differences between *Tylototriton sheni* sp. nov. and related species; Table S4: Between-group mean uncorrected p-distances among the sequences based on the 16S rRNA gene; Table S5: Between-group mean uncorrected p-distances among the sequences based on the *ND2* gene; Table S6: Comparison of *T*. *sheni* sp. nov. with other species of the *Tylototriton*.

## Appendix A

Specimens examined. *Tylototriton sheni* sp. nov., topotypes, 3 adult males, HUNL 2021042302, HUNL 201605007-08; 4 adult females, HUNL 201605005-06, 35, HUNL 2021042301. Collected from Zhongfang Co., Hunan, China; *Tylototriton maolanensis*, topotype, 1 adult male, HUNL 2025061601. Collected from Libo Co., Guizhou, China; *Tylototriton broadoridgus*, topotypes, 8 adult males, HUNL 19840513513, 527, 529, 532, HUNL 1982-859, HUNL 201605001-03; 4 adult females, HUNL 840513606, HUNL 1988-V-2, V-3, HUNL 201605004. Collected from Sangzhi Co., Hunan, China.
